# Adding Agnus Castus and Magnolia to Soy Isoflavones Relieves Sleep Disturbances Besides Postmenopausal Vasomotor Symptoms-Long Term Safety and Effectiveness

**DOI:** 10.3390/nu9020129

**Published:** 2017-02-13

**Authors:** Pasquale De Franciscis, Flavio Grauso, Anna Luisi, Maria Teresa Schettino, Marco Torella, Nicola Colacurci

**Affiliations:** Department of Woman, Child and General and Specialized Surgery—Second University of Naples, Largo Madonna delle Grazie, 1, 80138 Naples, Italy; flaviograuso@live.it (F.G.); anna-luisi@hotmail.it (A.L.); mariateresa.sche@gmail.com (M.T.S.); marco.torella@unina2.it (M.T.); nicola.colacurci@unina2.it (N.C.)

**Keywords:** isoflavones, menopause management, endometrial thickness, mammary density, liver function, plant extracts

## Abstract

The effectiveness for vasomotor symptoms and sleep disorders plus the long-term safety of a nutraceutical combination of *agnus-castus* and magnolia extracts combined with soy isoflavones (SI) and lactobacilli were assessed in postmenopausal women. A controlled study was carried out in menopausal women comparing this nutraceutical combination (ESP group) with a formulation containing isoflavones alone (C group) at the dosage recommended. The Kuppermann index, The Pittsburgh Sleep Quality Index (PSQI), and Short Form 36 (SF-36) were determined at baseline, three, six and 12 months. Endometrial thickness, mammary density and liver function were evaluated at baseline and after 12 months. One hundred and eighty women were enrolled in the study (100 in the ESP group and 80 in the C group). At the end of the treatment, mammary density, endometrial thickness, and hepatic function did not show substantial differences between groups. The Kuppermann index and particularly the tendency for hot flashes progressively and significantly decreased in frequency and severity during ESP versus C treatment. At the same time, a significant increase in sleep quality and psychophysical wellness parameters was observed in the ESP versus C groups. No adverse events were observed. *Agnus-castus* and magnolia, combined with SI + lactobacilli, can effectively and safely be used in symptomatic postmenopausal women, mainly when quality of sleep is the most disturbing complaint. The endometrium, mammary glands and liver function were unaffected after 12 months of treatment.

## 1. Introduction

Sleep disturbances negatively affect the quality of life of postmenopausal women; almost half of all menopausal women complain of sleep disorders, frequently associated with hot flashes and mood disturbances [1,2]. Hormone replacement therapy (HRT) is the first-line therapy in case of moderate/severe menopausal symptoms [3]; however, alternative treatments are needed in case of a contraindication to HRT, adverse side effects and poor compliance [4]. Moreover, many patients simply refuse HRT for a variety of reasons, mainly due to their fear of increasing the risk of cancer or weight gain [5], and prefer a “natural” approach [6].

Therapies based on soy isoflavones (SI) are the most popular approach: these substances have been shown to alleviate climacteric symptoms at a dose of between 40 and 80 mg/day [7]. Data on SI effects show that they exert elective stimulation of β-estrogen receptors (β-ERs) with less affinity and lower potency than estrogens [7] and stimulate the synthesis of sex hormone-binding globulin (SHBG) [8]. Furthermore, the β-ERs are poorly expressed in tissues which have a higher risk for estrogen-dependent carcinoma, and thus oncological safety could be expected with long-term use [9], even if data on the subject is still lacking [10,11].

The postmenopausal decrease of circulating estrogens is associated with typical vasomotor symptoms, among which the tendency for hot flashes is well studied. Even though sleep disturbances are poorly investigated, they are very common and significantly impair quality of life. Sleep complaints during menopause have been found in one in four to one in two of all women, as compared to approximately 15% of the general population [12]. They are also usually associated with mood disorders, particularly depression [13].

It is well known that populations with a traditionally soy-rich diet have a lower incidence and a lower intensity of vasomotor symptoms compared to postmenopausal women on a general diet [14]. Phytoestrogen consumption has been proposed as a mechanism for this difference and SI supplementation is used as an alternative to HRT for the treatment of climacteric symptoms in women with contraindications to HRT such as mammary cancer or advanced menopausal age [4,5]. However, the effect of SI is still far from being fully determined. While some studies suggest that hot flashes may benefit from SI administration [15], only little data has assessed the effect on sleep disturbances; in a pilot study, sleep complaints as observed by a polysomnographic analysis decreased from 90% to 37% in the treated group versus 95% to 63% in the placebo group [16]. To increase the clinical effectiveness, SI have been combined with different substances such as vitamins and oligominerals.

The strategy to add *agnus-castus* and magnolia to the SI formulation along with lactobacilli arises from the knowledge of their beneficial properties. *Agnus-castus* has been used in the treatment of many female conditions, including menstrual disorders (amenorrhea, dysmenorrhea), premenstrual dysphoric disorder (PMDD), corpus luteum insufficiency, hyperprolactinemia, infertility, acne, disrupted lactation, cyclic breast pain, cyclical mastalgia and inflammatory conditions, diarrhea and flatulence [17,18,19,20,21,22,23]. However, data regarding the use of *agnus-castus* during menopause is still lacking, despite early results showing its capabilities in reducing menopausal symptoms [24].

Magnolia has been shown to have tranquillizing and neurotrophic properties [25], but there are few reports investigating the effects on hot flashes, mood, and sleep symptoms [26]. Furthermore, it is shown that *agnus-castus* increases melatonin release, interacts with opioid receptors and can play a role in vasomotor symptoms and sleep diseases [7,25,27,28].

Furthermore, magnolia showed beneficial anxiolytic effects in premenopausal women [29]. Extracts of *Magnolia officinalis* bark and its active constituent, honokiol, have been studied in various mouse models which showed an activity similar to diazepam without the common side effects [29,30]. A recent study highlighted the efficacy of magnolia extract and magnesium on psycho-affective disorders and sleep disturbances in menopause, in addition to the effects of isoflavones on vasomotor symptoms [26].

SI have been combined with different oligoelements to enhance their clinical effects. It is reasonable to add SI with lactic acid bacteria in the form of spores, resistant to the gastric and biliary secretion, to promote the action of bacterial glycosidase and to assure the bioavailability of SI [31,32]. It is well known that the absorption of SI can differ greatly between patients as it depends on the activity of the glucosidases of intestinal microflora in the lower bowel. In fact, glucosidases could liberate the aglycones from the glucosides and promote absorption of SI [33,34].

Data regarding the influence of SI on sleep disturbances is conflicting [7,35].

It is on this basis that we have analyzed the effectiveness and safety of long-term therapy using *agnus-castus* and magnolia combined with SI and lactobacillus on vasomotor symptoms, in particular focusing on sleep disorders in menopausal women.

## 2. Materials and Methods

In a prospective, observational, controlled study, 180 postmenopausal patients in non-hormonal treatment for menopausal vasomotor symptoms gave their informed consent to participate. The patients were monitored for one year.

The study was approved by the Ethical committee of Second University of Naples (Protocol Number 138 of 19 February 2014) and was carried out according to the principles of Helsinki Declaration.

Inclusion criteria were age between 45 and 65 years, Follicle-Stimulating Hormone (FSH) >30 mUI/mL, Estradiol (E2) <20 pg/mL; Kupperman score >20 and <30, sleep disorders score >5, non-hormonal therapy initiated by less than 30 days.

Enrolled patients were divided into two groups for treatment:

- Group 1: the ESP group (100 women) received one tablet/day containing SI 60 mg, *Lactobacillus sporogenes* 109 spores, *Magnolia officinalis* extract 50 mg, *Vitex agnus-castus* extract 40 mg and vitamin D 35 μg. This combination is commercially available in one tablet.
- Group 2: the C group (80 women) received one tablet/day containing only isoflavones 60 mg.

The exclusion criteria were soy-enriched diet, Body Mass Index (BMI) >30, breast- or endometrial diseases, inability to understand the study finalities and to give informed consent.

The treatment efficacy was evaluated at baseline and every three months by:

(a) The Kupperman index. The score is the result of a self-completed questionnaire that evaluates frequency and subjective intensity of 11 among the most frequent vasomotor symptoms in postmenopausal women (hot flushes, insomnia, irritability, sweating, musculoskeletal pains, headache, palpitations, fatigue, paresthesia). The symptoms were rated as mild (score 15–20), moderate (20–35), severe (>35). Number and daily intensity of hot flushes were recorded on a self-completed diary (0 = absent; 1 = mild; 2 = moderate; 3 = severe; 4 = very severe).
(b) The Pittsburgh Sleep Quality Index (PSQI) test for the evaluation of sleep disorders. It is a 19-item questionnaire regarding the last month’s symptomatology. The questionnaire generates seven composite scores, on a scale from 0 to 3; so the PSQI global score is from 0 to 21. Higher scores reflect more severe sleep disorders (the cut-off for sleep disturbances is a score of >5). The results give numbers in seven categories: subjective sleep quality, sleep latency, sleep duration, habitual sleep efficiency, sleep disturbances, use of sleeping medication, and daytime dysfunction (26).
(c) Short Form 36 (SF-36). It is a test submitted at baseline and after one year to evaluate the psycho-physics wellness parameters. The SF-36 health survey consists of 36 questions evaluating functioning and well-being. Each of the questionnaire items refers to one of the following eight different health indicators: physical functioning; physical-role, referred to the limitations in performing relevant life roles due to physical health; bodily pain; general health; vitality; social functioning; limitations in performing relevant life roles due to emotional problems; mental health, referring to the absence of anxiety and depression. The test gives results in eight composite scores and a total score on a scale from 0 to 100. Lower scores show more severe impairment of psychophysics wellness parameters.

Safety of treatment was assessed at baseline (T0) and after one year. It was based on endometrial thickness evaluated by trans-vaginal ultrasonography, mammographic density and hepatic function assessed by transaminases, bilirubin, γ-glutamyltransferase (γ-GT).

Statistical analysis was carried out using *t*-test of Student for paired and non-paired data, the Mann-Whitney test for the non-parametric analysis. The Wilcoxon matched-pairs test was used for inside and between groups’ comparison and Chi-square test to compare the frequencies. Statistical Package for Social Sciences (SPSS) 10.0 (National Opinion Research Center, Chicago, IL, USA) was the software.

## 3. Results

The clinical features of patients enrolled in the study are reported in Table 1 according to the treatment group.

After 12 months of treatment, the mammographic density, endometrial thickness and hepatic function did not show significant differences between the ESP and C groups (Table 2).

**Figure 1:**
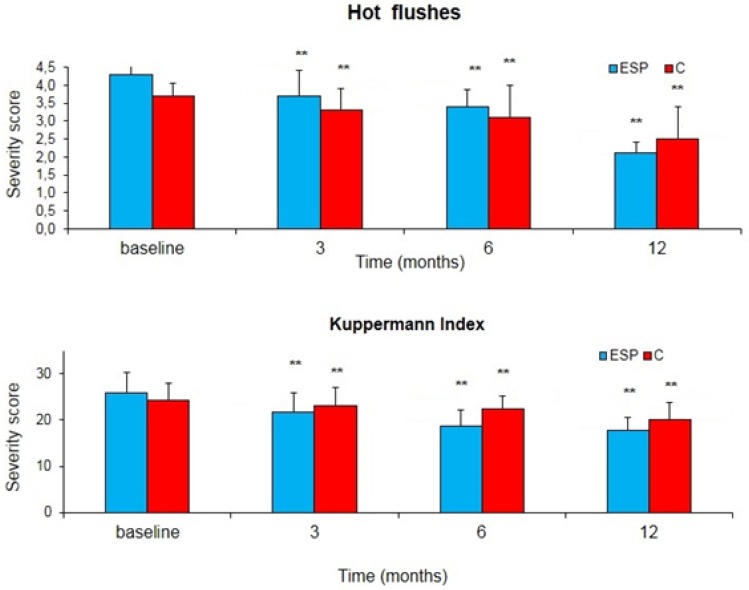
Changes in the severity score of hot flashes and the Kuppermann index during the treatment with soy isoflavones + lactobacilli + magnolia + agnus castus (ESP) or isoflavones alone (C). Data is expressed as mean ± Standard Error (SE). ** *p* < 0.01 vs. baseline.

Concerning the clinical effectiveness, we observed a significant decrease in hot flashes and the intensity of symptoms in the ESP group, as shown in Figure 1. After 12 months, the hot flash severity score significantly decreased from 4.31 to 2.12 in the ESP group (−50.8%), while it only decreased from 3.7 to 2.5 in the C group (−32.4%) (*p* < 0.01); the Kuppermann index severity score significantly decreased from 25.9 to 17.8 in the ESP group (−31.3%) and only from 24.3 to 20.1 in the C group (−17.3%) (*p* < 0.01).

SF-36 significantly improved in the ESP group from 38.0 ± 5.8 to 52.5 ± 6.3 (+38.2%), while it remained unchanged in the C group, from 56.0 ± 5.7 to 51.0 ± 7.3 (−8.9%) (*p* < 0.01).

**Figure 2:**
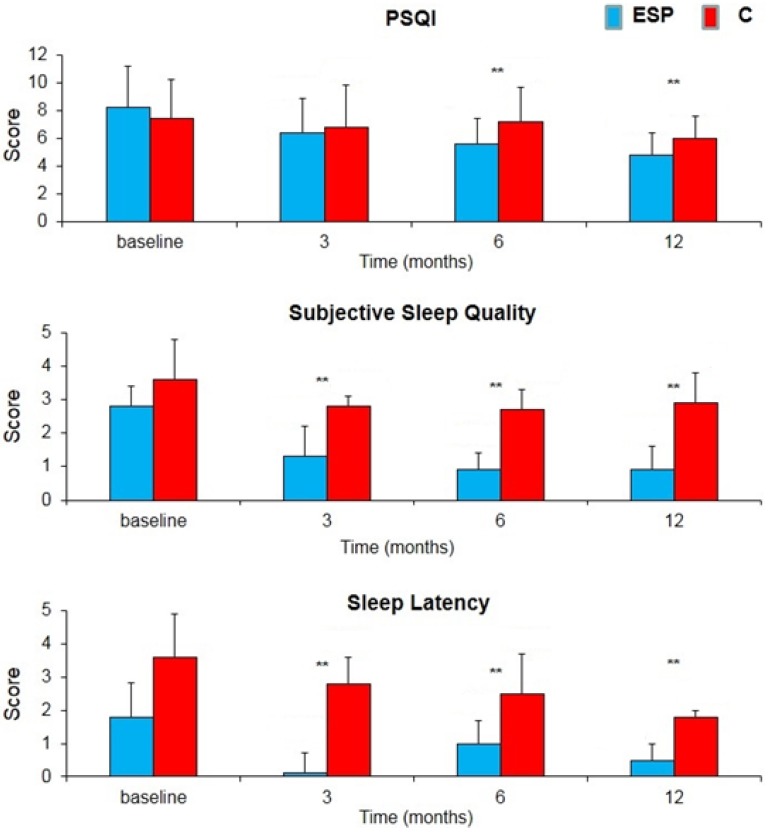
Changes in severity score of The Pittsburgh Sleep Quality Index (PSQI), subjective sleep quality and sleep latency during the treatment with soy isoflavones + lactobacilli + magnolia + agnus castus (ESP) or isoflavones alone (C). Data is expressed as mean ± SE. ** *p* < 0.01 vs. baseline.

A significant increase in sleep quality and psychophysical wellness parameters was observed, particularly concerning the better subjective quality of sleep and a better ability to fall asleep in the ESP vs. C groups (Figure 2). After 12 months the PSQI score significantly decreased from 8.3 to 4.8 in the ESP group (−42.2%) and only from 7.5 to 6.0 in the C group (−20.0%) (*p* < 0.01); The subjective sleep quality score significantly decreased from 2.8 to 0.9 in the ESP group (−67.9%) and only from 3.6 to 2.9 in the C group (−19.4%) (*p* < 0.01). The Sleep latency score significantly decreased from 1.8 to 0.5 in the ESP group (−72.2%) and only from 3.6 to 1.8 in the C group (−50.0%) (*p* < 0.01).

In fact, the high scores of PSQI parameters significantly decreased in ESP group more than C group.

## 4. Discussion

In the present study, a combination of 60 mg SI with *Lactobacillus sporogenes*, *agnus-castus* and magnolia was compared to SI alone. The presence of lactobacilli allows us to guarantee the intestinal absorption and bioavailability of the SI [31,33], but it could theoretically enhance the estrogenic effects and the risk of hormone-dependent tumors [36]. The results are reassuring as regards both effectiveness and safety. A statistically significant decrease in the number and intensity of hot flashes was observed in the Kupperman index in the ESP group compared to the SI group. Neither endometrial stimulation nor an increase of breast density was observed. A significant part of the beneficial effect was observed during the first three to six months of therapy: such data are relevant for the patient’s acceptance and the guarantee of long-term compliance. The improvement of symptoms was confirmed after one year of therapy.

Significant vasomotor symptom relief was recorded in the ESP group vs. the C group. Indeed, the improvement of global sleep quality was more evident in the ESP versus C group as shown by the lower score in the PSQI test. Such an improvement in sleep quality is related to the action of magnolia and *agnus-castus* extracts in addition to the decrease in hot flashes occurring during the night. As is well known, magnolia and *agnus-castus* have neurotrophic and anxiolytic properties, explaining the positive effect on symptoms such as irritability, melancholy, headache, and tiredness. A beneficial effect on these symptoms is associated with a better quality of sleep, in particular subjective sleep satisfaction, less nocturnal awakening and lower nightmare frequency. The population taking the treatment showed better subjective sleep quality and an easier ability to fall asleep, resulting in a better quality of life as shown by the SF-36 test results.

This study has the advantage that a suitable number of patients were followed for an appropriate period of time so as to confer statistical significance to the results. However, this study has its limits: the use of a single tablet containing a combination of magnolia, *agnus-castus*, vitamin D3 and *Lactobacillus sporogenes* in the ESP group did not elucidate the contribution that each individual component made to the end result. Further studies using even larger populations over longer periods of time with investigations of the individual components would be helpful.

In conclusion, *agnus-castus* and magnolia in combination with SI can effectively and safely be used in the treatment of vasomotor symptoms in postmenopausal women, especially when quality of sleep is the most disturbing complaint.

## Figures and Tables

**Table 1:** Anamnestic and clinical features.

	ESP Group	C Group
Age (Years)	55.0 ± 6.4	56.0 ± 5.8
BMI	24.9 ± 2.9	25.7 ± 4.3
Period of Amenorrhea (Years)	3.2 ± 0.8	2.6 ± 0.4

*p* value = 0.99; BMI: Body Mass Index.

**Table 2:** Safety profile.

	Group ESP (*n* = 100)		Group C (*n* = 80)	
	Baseline	12 Months	Baseline	12 Months
Mammographic density (%)	1.80 ± 0.7 *	1.86 ± 0.8 **	1.75 ± 0.7 *	1.79 ± 0.6 **
Endometrial thickness (mm)	3.4 ± 0.9 *	3.2 ± 0.6 **	3.8 ± 1.2 *	3.3 ± 1.1 **
ALT (UI/L)	18.7 ± 6.5 *	20.2 ± 4.2 **	19.7 ± 2.6 *	21.3 ± 3.8 **
AST (UI/L)	21.4 ± 5.3 *	20.3 ± 6.2 **	19.2 ± 4.6 *	19.8 ± 3.4 **
γGT (UI/L)	24.3 ± 15.6 *	22.6 ± 19.3 **	20.4 ± 17.3 *	24.8 ± 18.2 **
Bilirubin (mg/dL)	0.46 ± 0.22 *	0.40 ± 0.26 **	0.48 ± 0.34 *	0.42 ± 0.23 **

* *p* value = 0.97; ** *p* value = 0.99; ALT: Alanine Aminotransferase; AST: aspartate aminotransferase; γ-GT: γ-glutamyltransferase.
